# Quality of Life of Cancer Patients Receiving Enteral Nutrition: A Systematic Review of Randomized Controlled Trials

**DOI:** 10.3390/nu13124551

**Published:** 2021-12-19

**Authors:** Elwira Gliwska, Dominika Guzek, Zuzanna Przekop, Jacek Sobocki, Dominika Głąbska

**Affiliations:** 1 Department of Food Market and Consumer Research, Institute of Human Nutrition Sciences, Warsaw University of Life Sciences (WULS-SGGW), 159C Nowoursynowska Street, 02-776 Warsaw, Poland; elwira_gliwska@sggw.edu.pl; 2 Cancer Epidemiology and Primary Prevention Department, The Maria Sklodowska-Curie National Research Institute of Oncology, 15B Wawelska Street, 02-034 Warsaw, Poland; 3 Department of Clinical Dietetics, Faculty of Health Sciences, Medical University of Warsaw, 27 Ciołka Street, 01-445 Warsaw, Poland; zuzanna.przekop@wum.edu.pl; 4 Department of General Surgery and Clinical Nutrition, Centre of Postgraduate Medical Education in Warsaw, 231 Czerniakowska Street, 00-416 Warsaw, Poland; jsobocki@mp.pl; 5 Department of Dietetics, Institute of Human Nutrition Sciences, Warsaw University of Life Sciences (WULS-SGGW), 159C Nowoursynowska Street, 02-776 Warsaw, Poland; dominika_glabska@sggw.edu.pl

**Keywords:** cancer, diet, nutrition, enteral nutrition, oncology, quality of life, QoL, randomized controlled trials

## Abstract

Most studies confirm the beneficial effects of enteral nutrition on the quality of life, but some studies indicate an inverse association and its detrimental impacts. However, there are insufficient data on the effects of enteral nutrition on the quality of life of cancer patients. This systematic review aimed to describe the influence of applied enteral nutrition on the quality of life of cancer patients, based on the results of randomized controlled trials. It was registered in the PROSPERO database (CRD42021261226) and conducted based on the PRISMA guidelines. The searching procedure was conducted using the PubMed and Web of Science databases, as well as Cochrane Library, and it included studies published until June 2021. It was conducted to select randomized controlled trials assessing the influence of enteral nutrition (compared with the other model of nutrition) on the quality of life of cancer patients. A general number of 761 records were screened and a final number of 16 studies were included in the systematic review. The studies were included and assessed by two independent researchers, while the risk of bias was analyzed using the Newcastle–Ottawa Scale (NOS). Studies compared patients treated with and without enteral nutrition, patients treated with various methods of enteral nutrition or with enteral diets of various content, as well as patients treated with enteral and parenteral nutrition. Within the included studies, the majority were conducted in patients with cancers located in various parts of the body, or diverse areas within the gastrointestinal system, while some studies were conducted in specific populations of patients with a defined cancer location—esophagus, stomach, or ovary. The duration of applied enteral nutrition within the included studies was diversified—from two weeks or less to half a year or even more. The vast majority of studies used well-known and validated tools to assess the quality of life, either developed for a specific group of head/neck, esophagus/stomach, and ovary cancer patients or developed for more general patient populations. Most studies concerning patients treated with and without enteral nutrition supported applying enteral nutrition, which was concluded in seven studies out of ten (including four studies with a low risk of bias). The other important observations to be emphasized—formulated based on the studies with a low risk of bias—presented the role of oral supportive nutrition guided by a dietitian, as well as the beneficial role of enteral and parenteral nutrition, combined. In spite of a relatively low number of randomized controlled trials assessing the influence of enteral nutrition on the quality of life of cancer patients, which should be considered as a limitation, the results were promising. Most studies supported the positive influence of enteral nutrition on the quality of life, either assessed based on the psychological measures of the quality of life or by considering the other potential determinants (e.g., malnutrition, complications, etc.). Taking this into account, enteral nutrition should be applied whenever possible, both to prevent and treat malnutrition in cancer patients. However, considering the limited number of studies conducted so far, further research conducted in homogenic populations of patients is necessary.

## 1. Introduction

Cancer is a growing global problem, being the first or second leading cause of death of individuals aged under 70 years in 112 of 183 countries, according to the World Health Organization (WHO) [1]. The Global Cancer Observatory (GCO) by the International Agency for Research on Cancer (IARC) and the WHO, within their GLOBOCAN 2020 estimates of incidence and mortality, indicated nearly 19.3 million new cancer cases and almost 10 million cancer deaths registered worldwide in 2020 [2]. Taking this into account, the WHO emphasizes that cancer is one of the main challenges for public health within both areas of prevention and treatment [3].

The cancer treatment methods are classified by the National Cancer Institute (NCI) as biomarker testing, chemotherapy, hormone therapy, immunotherapy, radiation therapy, stem cell transplant, surgery, and targeted therapy [4]. As indicated in the systematic review by Shrestha et al. [5], while choosing the therapeutic option, the length of life and quality of life are taken into consideration—patients with better health value rather than length of life over quality of life, and those with poorer physical status value rather than the quality of life over the length of life. The quality of life is defined as a sense of well-being and includes physical, psychological, social, and spiritual aspects, which may be changed in cancer patients [6]. The quality of life of cancer patients is significantly reduced [7,8,9], which results from the disease process itself— its course, symptoms and complications, the applied treatment, and the disease duration [10]. 

Among cancer symptoms and complications, malnutrition is one of the most common, as it results from anorexia and metabolic dysregulation combined, both caused by the tumor itself or by its treatment and contributing to cachexia [11]. It may affect up to 80% of cancer patients, while its prevalence depends on the cancer type, disease setting, comorbidities, and type of treatment performed [12]. Although the problem of malnutrition and cancer-related cachexia have been known for a long time, effective prevention and treatment remain a challenge [13]. Prevention and treatment are especially important as malnutrition not only affects the effectiveness of cancer treatment, as well as the prognosis and hospital stay length [14], but also influences the quality of life [15,16,17]. 

Taking this into consideration, the European Society for Clinical Nutrition and Metabolism (ESPEN), within its guidelines, indicated that the most important action against cancer-related malnutrition is to provide early screening and to assure individualized nutritional interventions [18]. An effective, personalized nutrition plan should include not only an appropriate diet or oral nutrition support but also enteral or parenteral nutrition if needed [19]. However, the recommendations by ESPEN indicate the superiority of feeding by the gastrointestinal tract over parenteral nutrition, and enteral nutrition is recommended if possible [20]. Similarly, the systematic review by Chow et al. [21] indicated that, for cancer patients, parenteral nutrition may result in an increased risk of complications compared with enteral nutrition but would not prolong survival.

However, there are insufficient data on the effects of enteral nutrition on the quality of life of cancer patients. A recent systematic review by Ojo et al. [22] assessed the effect of enteral tube feeding on the quality of life of various patients, including not only cancer patients but also those with other diseases and conditions. Based on this review, it was stated that most studies confirm the beneficial effect of enteral nutrition, but some studies indicate inverse association and its detrimental effects on the quality of life [22]. Taking this into account, the present systematic review aimed to describe the influence of applied enteral nutrition on the quality of life of cancer patients, based on the results of randomized controlled trials.

## 2. Materials and Methods

### 2.1. The Systematic Review Design and Registration

The systematic review of the influence of applied enteral nutrition on the quality of life in cancer patients was based on the Preferred Reporting Items for Systematic Reviews and Meta-Analyses (PRISMA) recommendations for the literature search and screening, including studies and reporting results [23]. The literature search was based on the PubMed and Web of Science databases, as well as Cochrane Library, and included studies published until June 2021.

The systematic review was registered in the International Prospective Register of Systematic Reviews (PROSPERO) database (CRD42021261226). 

### 2.2. The Inclusion and Exclusion Criteria

The studies that were assessed within the present systematic review were intended to be randomized controlled trials assessing the influence of enteral nutrition (compared with the other model of nutrition) on the quality of life of cancer patients.

The inclusion criteria comprised research articles, presenting randomized controlled trials with full texts published in peer-reviewed journals in English, as well as the studied populations of cancer individuals with any enteral nutrition applied and quality of life assessed in any way.

The exclusion criteria comprised studies conducted in an animal model, studies not comparing enteral nutrition with any other model of nutrition but assessing technical aspects of enteral nutrition (such as the study by Patel et al. [24]), and studied populations of participants with any eating disorder (influencing the effectiveness of enteral nutrition applied) or intellectual disability (influencing the declared quality of life assessed within the studies).

While including the studies, no additional criteria associated with the type of cancer, characteristics of the studied population, or country were taken into account.

The summarized inclusion and exclusion criteria for the patient, intervention/exposure, comparator, outcome, and study design (PICOS) are presented in Table 1.

### 2.3. The Procedure of Systematic Review

The electronic search was conducted within the PubMed and Web of Science databases, as well as Cochrane Central Register of Controlled Trials, and the detailed electronic search strategy is presented in Table 2. 

**Figure 1:**
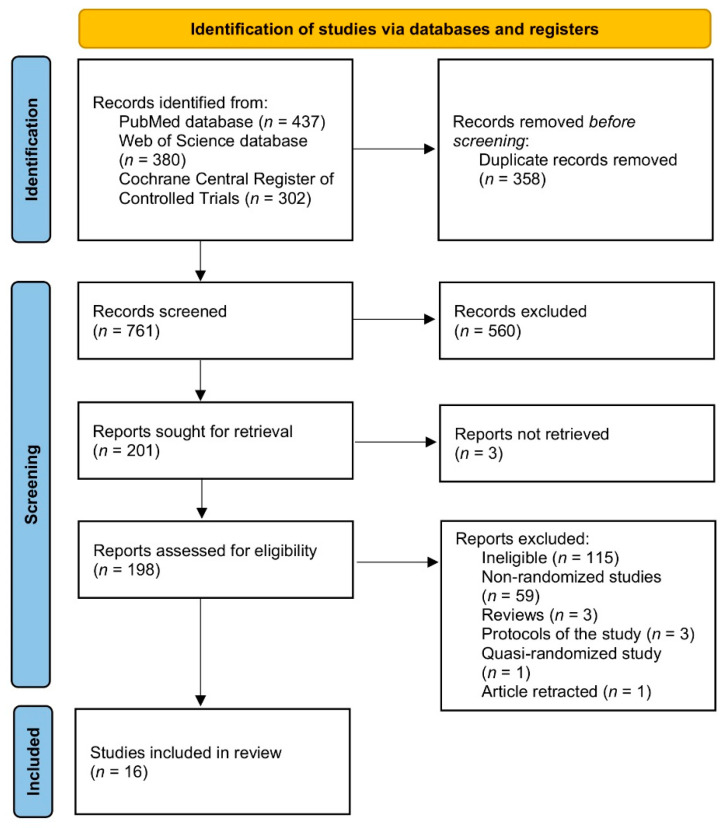
The procedure of identification, screening, and inclusion applied within the systematic review.

The procedure of identification, screening, and inclusion applied within the systematic review is presented in Figure 1. The identification of the eligible studies was performed independently by two researchers and conducted within three stages—based on the title, abstract, and full text of the study. The titles and abstracts were sourced from electronic databases, and full texts were also sourced from corresponding authors of studies if in electronic databases they were unavailable. If any disagreement appeared at any stage, it was consulted with the other researcher.

### 2.4. The Procedure of Data Extraction

Data extraction was performed independently by two researchers and if any disagreement appeared, it was consulted with the other researcher. If any information was unavailable within the study, it was obtained from the corresponding author of the study and, in such cases, it is referred to as data provided on request.

The extracted data comprised basic characteristics of the study (study design as defined within the article, the country with detailed location, and the time when the study was conducted), basic characteristics of the studied influence (nature of the studied group as defined within the study, disease location, and psychological measure for the assessed quality of life), basic characteristics of the studied group (the number of studied participants and female participants, age of the studied group, inclusion criteria, and exclusion criteria), basic characteristics of the applied nutritional intervention associated with enteral nutrition (applied enteral nutrition, duration of applied nutritional intervention, and any other information about nutrition as defined within the article), and the results and conclusions (the effect of the applied nutritional intervention on the quality of life).

The assessment of the risk of bias was conducted to define the methodological quality of the included studies [25] and the Newcastle–Ottawa Scale (NOS) [26] was used. The selection, comparability, and exposure/outcome were scored as follows: 0–4, 0–2, and 0–3 and, afterward, the total score was attributed to the categories of very high risk of bias (total score of 0–3), high risk of bias (total score of 4–6), and low risk of bias (total score of 7–9) [27]. 

## 3. Results

The basic characteristics of the studies included in the systematic review [28,29,30,31,32,33,34,35,36,37,38,39,40,41,42,43] are presented in Table 3. Within the included studies, the majority were conducted in European countries—in Sweden [29,32,33,34], United Kingdom [35,39], Netherlands [28], Italy [38] and Poland [42]—but there were also some studies conducted in China [37,40,43] and Australia [36,41], which may have influenced various approaches applied to enteral nutrition. Most of the studies were conducted in the 2010s [31,35,36,37,38,39,40,41,43], but there were also some studies conducted in the 2000s [30,31,32,33,34,36,38] and even the 1990s [28].

The basic characteristics of the influence studied within the studies included in the systematic review are presented in Table 4. The majority of the included studies were conducted in patients with cancers in various locations [28,30,31,32,33,34,41,42] such as the esophagus [40,43], stomach [37], or ovary [36], or various locations within the gastrointestinal system [29,35,38,39]. Most of the studies presented populations recruited from a specific hospital/clinic/department [28,29,31,33,35,37,39,40,41,42,43] or a cancer registry [32,34,38], but for some studies, the specific clinic of origin was not defined [30,36]. The vast majority of studies used well-known and validated tools to assess the quality of life, either developed for a specific group of head/neck [31,32,33,34,41], esophagus/stomach [35,39], and ovarian cancer patients [36], or developed for more general populations of patients [28,29,32,34,36,37,38,39,40,41,42,43]. Only one study used a tool that was not previously validated and was developed based on other validated tools [30].

The basic characteristics of the groups studied within the studies included in the systematic review are presented in Table 5. The included studies presented the observations formulated in a samples of various sizes—small samples of less than 50 participants [28,30,35,39,42], medium-size samples of less than 100 participants [29,37,38,40,43], or large samples of 100 or more participants [31,32,33,34,36,41]. The age of the studied patients in most studies was about 60 years [28,29,30,32,33,34,35,36,37,39,41,42,43], but there were also some studies analyzing younger patients of less than 60 [40] or older patients of more than 60 years [38]. Within the inclusion criteria, the diagnosis of cancer was considered [28,29,30,31,32,33,34,35,36,37,38,39,40,41,42,43], but in some studies, it was accompanied by malnutrition [28,30,36,38].

The basic characteristics of the nutritional interventions associated with enteral nutrition applied within the studies included in the systematic review are presented in Table 6. The included studies compared patients treated with and without enteral nutrition [28,32,33,34,35,36,38,39,41,43], patients treated with various methods of enteral nutrition [30,31] or with enteral diets of various content [42], and patients treated with enteral and parenteral nutrition [29,37,40]. The duration of applied enteral nutrition within the included studies was diversified—from two weeks or less [28,40], through a few weeks or months [30,31,35,36,38,39,42,43], to half a year or even more [29,32,33,34].

The results and conclusions associated with the effect of the applied nutritional intervention on the quality of life within the studies included in the systematic review are presented in Table 7. The results presented are based on the description formulated by authors of the studies within their abstracts.

The summary of conclusions from the studies comparing patients treated with and without enteral nutrition included in the systematic review accompanied by the Newcastle-Ottawa Scale (NOS) score is presented in Table 8. It was stated that the majority of studies supported applying enteral nutrition, which was concluded in seven studies out of ten (including four studies of a low risk of bias). Although two of the supporting studies [32,34] were conducted within the same cohort, such observation is prominent. 

The summary of conclusions from the studies comparing patients treated with enteral and parenteral nutrition, with various methods of enteral nutrition, and with enteral nutrition of various contents, included in the systematic review accompanied by the Newcastle–Ottawa Scale (NOS) score is presented in Table 9. The important conclusions that should be emphasized, formulated based on the studies of a low risk of bias, present the role of oral supportive nutrition guided by a dietitian [29], as well as the beneficial role of enteral and parenteral nutrition combined [40].

## 4. Discussion

Due to an increase in the effectiveness of anti-cancer treatment [44] and an increase in life expectancy in cancer patients [45], the long-term complications will probably be observed more often, resulting in increasing role of the quality of life [46]. Taking this into account, it must be emphasized that the systematic review by Lis et al. [47], assessing the role of nutritional status in predicting quality of life in cancer individuals, indicated that correcting malnutrition may improve quality of life in cancer patients. Considering this, the presented systematic review is based on the assumption that enteral nutrition may promote a better nutritional status.

In agreement with the indicated association between nutritional status and quality of life, the ESPEN, within its recent practical guidelines [48] recommended applying nutritional support, including dietary advice, oral nutrition supplements, and enteral and parenteral nutrition as an effective way of improving nutritional status and malnutrition prevention. However, while choosing the method of nutritional support, it is indicated that, despite nutritional interventions, enteral nutrition should be recommended if oral nutrition remains inadequate, and parenteral nutrition should be recommended if enteral nutrition is not sufficient or feasible [48].

The results of the conducted systematic review of the randomized controlled trials confirmed the beneficial effects of enteral nutrition for cancer patients in the area of quality of life. While comparing patients treated with and without enteral nutrition, it was stated that enteral nutrition has a beneficial effect on the quality of life in a majority of studies, confirmed in groups of head and neck cancer patients [28,32,34], upper gastrointestinal tract cancer patients [38,39,43], and ovarian cancer patients [36]. At the same time, the results were not so consistent while comparing patients treated with enteral and parenteral nutrition; depending on the study, the various results were observed [29,37,40], but generally combined enteral and parenteral nutrition was stated to be superior to both enteral [40] and parenteral nutrition alone [37]. The indicated observations are in agreement with the recommendations by ESPEN [48], indicating the need to meet the energy requirements of patients, which must be considered the overall objective.

In spite of the fact that the majority of included studies concluded the beneficial role of enteral nutrition (especially while compared with no nutritional support), some disadvantages or contradictory results are also indicated. Such observations were formulated mainly within studies assessing the effect of prophylactic enteral nutrition, applied, not when necessary, but earlier, in order to limit the risk of malnutrition [33,35,41]. This may result from the fact that the enteral nutrition procedure itself can generate complications [49]. As such complications may indirectly affect the quality of life, each of them needs to be considered while choosing the best option for nutritional support.

While describing the results gathered within randomized controlled trials, it should be emphasized that only a small number of such studies have been conducted so far, while various methods of enteral nutrition and various nutritional values of diet have been studied. The various studies comparing patients treated with and without enteral nutrition and comparing patients treated with enteral and parenteral nutrition are incomparable due to various enteral nutrition procedures applied within the studies. At the same time, only two randomized controlled trials comparing patients treated with various methods of enteral nutrition [30,31], and one comparing patients treated with enteral nutrition of various contents [42], have been conducted so far, so no deeper conclusions can be made, especially if the results of the studies are contradictory [30,31].

The other problem while comparing the results of the included studies is associated with various cancer locations in the included studies, but also with the well-known diverse cancer course and intra-patient variability observed in the treatment effectiveness [50]. As a result, gathering and combining results observed within various studies may be impossible. 

Despite the described difficulties in synthesizing results of the included studies, the most prominent observation formulated within the majority of studies remains consistent and is associated with the positive influence of enteral nutrition on the quality of life. While the quality of life is linked to the stage of cancer [51], the prognosis [52], malnutrition [53], and applied therapy [54], enteral nutrition must also be taken into account as a factor indirectly affecting it by improving the effectiveness of cancer therapy [55] and reducing the risk of malnutrition [56].

Considering the described results of gathered randomized controlled trials, and in agreement with the ESPEN guidelines, enteral nutrition should be applied whenever possible to improve the quality of life of cancer patients.

## 5. Conclusions

Most of the studies support the positive influence of enteral nutrition on the quality of life, either assessed based on the psychological measures of the quality of life or by considering the other potential determinants (e.g., malnutrition, complications, etc.). Taking this into account, enteral nutrition should be applied whenever possible, both to prevent and treat malnutrition in cancer patients. However, considering the limited number of studies conducted so far, further research conducted in homogenic populations of patients is necessary.

## Figures and Tables

**Table 1:** The summarized inclusion and exclusion criteria for a patient, intervention/exposure, comparator, outcome, and study design (PICOS).

PICOS	Inclusion Criteria	Exclusion Criteria
Population	Cancer patients	Animal models; patients with eating disorders or intellectual disabilities
Intervention/exposure	Enteral nutrition applied	Enteral nutrition not described within the study
Comparison	Influence of enteral nutrition on the quality of life	Lack of comparison of enteral nutrition with the other model of nutrition
Outcome	Quality of life assessed	Quality of life not presented within the study
Study design	Randomized controlled trials	Articles not published in English, not published in peer-reviewed journals, retracted articles

**Table 2:** The detailed electronic search strategy applied within the systematic review for the PubMed and Web of Science databases, as well as Cochrane Central Register of Controlled Trials.

Database/Register	Detailed Electronic Search Strategy
PubMed	(“cancer”[Title/Abstract] OR “tumor”[Title/Abstract] OR “oncology”[Title/Abstract]) AND (“quality of life”[Title/Abstract] OR “QoL”[Title/Abstract]) AND (“enteral”[Title/Abstract] OR “enteric”[Title/Abstract] OR “intragastric”[Title/Abstract] OR “intraintestinal”[Title/Abstract] OR “intestinal”[Title/Abstract] OR “tube”[Title/Abstract]) AND (“nutrition”[Title/Abstract] OR “feeding”[Title/Abstract])
Web of Science	AB = (cancer OR tumor OR oncology) AND AB = (quality of life OR QoL) AND AB = (enteral OR enteric OR intragastric OR intraintestinal OR intestinal OR tube) AND AB = (nutrition OR feeding)
Cochrane Library	(“cancer” OR “tumor” OR “oncology”):ti,ab,kw AND (“quality of life” OR “QoL”):ti,ab,kw AND (“enteral” OR “enteric” OR “intragastric” OR “intraintestinal” OR “intestinal” OR “tube”):ti,ab,kw AND (“nutrition” OR “feeding”):ti,ab,kw

**Table 3:** The basic characteristics of the studies included in the systematic review.

Ref.	Authors, Year	Study Design	Country/ Detailed Location	Time
[28]	van Bokhorst-de van der Schueren et al., 2000	Randomized clinical trial	Netherlands/Amsterdam	1994–1997
[29]	Hyltander et al., 2005	Randomized study	Sweden/Göteborg	Lack of data
[30]	Corry et al., 2008	Randomized study	Lack of data	2000–2002
[31]	Sadasivan et al., 2012	Prospective, randomized, controlled study	Lack of data	2009–2011
[32]	Silander et al., 2012	Randomized study	Sweden/Göteborg	February 2002–December 2006
[33]	Silander et al., 2013	Randomized longitudinal study	Sweden/Göteborg	February 2002–December 2006
[34]	Axelsson et al., 2017	Randomized controlled study	Sweden/Göteborg	2002–2010
[35]	Bowrey et al., 2015	Randomized controlled trial	United Kingdom/Leicester	July 2012–September 2014
[36]	Baker et al., 2015	Randomized trial	Australia/Queensland	2009–2013
[37]	Li et al., 2015	Double blind placebo trial	China/Xinxiang	May 2012–May 2014
[38]	Gavazzi et al., 2016	Multicenter, controlled, open-label, randomized clinical trial	Italy/Milan	December 2008–June 2011
[39]	Froghi et al., 2017	Randomized trial	United Kingdom/Devon	December 2012–July 2014
[40]	Wu et al., 2017	Open, randomized, controlled trial	China/Shanghai	Inclusion: July 2012–January 2013
[41]	Brown et.al., 2017	Randomized controlled trial	Australia/Queensland	September 2012–August 2016
[42]	Kaźmierczak-Siedlecka et al., 2020	Double-blind, randomized placebo-controlled trial	Poland/Gdańsk	Lack of data
[43]	Liu et al., 2020	A pilot parallel-group, randomized single-blind, clinical trial	China/Nanjing	Inclusion: January–June 2018

**Table 4:** The basic characteristics of the influence studied within the studies included in the systematic review.

Ref.	Studied Group	Disease Location	Psychological Measure
[28]	Severely malnourished head and neck cancer patients eligible for surgery from the Department of Otolaryngology/Head and Neck Surgery of the Vrije Universiteit, Academic Hospital	Oral cavity, oropharynx, hypopharynx, larynx, other	EORTC QLQ-C30; COOP-WONCA charts
[29]	Upper gastrointestinal tract cancer patients undergoing resections in Department of Surgery at the Sahlgrenska University Hospital	Esophagus, stomach, pancreas	Eating Dysfunction Scale; Gastrointestinal Symptom Rating Scale; PGWBI; EORTC QLQ-C30
[30]	Head and neck squamous cell carcinoma patients	Oral cavity, oropharynx, nasopharynx, hypopharynx, larynx, other	The non-validated questionnaire developed based on EORTC QLQ-H&N35
[31]	Head and neck cancer patients from the Otorhinolaryngology Department	Hypopharynx, larynx, nasopharynx, oropharynx	EORTC QLQ-H&N35
[32]	Newly diagnosed advanced head and neck cancer patients randomized at the Regional Cancer Registry	Oropharynx, oral cavity, hypopharynx, nasopharynx, other	EORTC QLQ-C30; EORTC QLQ-H&N35
[33]	Newly diagnosed advanced head and neck cancer patients in the Department of Otorhinolaryngology—Head and Neck Surgery at the Sahlgrenska University Hospital	Oral cavity, pharynx, neck lymph node	EORTC QLQ-H&N35—swallowing sub-scale
[34]	Newly diagnosed advanced head and neck cancer patients randomized at the Regional Cancer Registry	Oropharynx, oral cavity, hypopharynx, nasopharynx, other	EORTC QLQ-C30; EORTC QLQ-H&N35
[35]	Esophageal or gastric cancer patients from University Hospitals of Leicester NHS Trust Oesophagogastric Cancer Service	Esophagus, stomach	EORTC QLQ-C30; EORTC QLQ-OG25; EQ-5D-3L
[36]	Advanced epithelial ovarian cancer patients	Ovary	FACT-G; FACT-O; EQ-5D VAS; EQ-5D
[37]	Gastric cancer patients from the Department of General Surgery, Xinxiang Central Hospital	Stomach	SF-36
[38]	Upper gastrointestinal tract cancer patients from Fondazione IRCCS Istituto Nazionale dei Tumori and at the European Institute of Oncology	Esophagus, pancreas, stomach, biliary tract	FAACT
[39]	Patients from Peninsula Oesophago-Gastric Unit, Derriford Hospital undergoing upper gastrointestinal surgery for cancer	Esophagus, stomach	MFI-20; EQ-5D; EORTC QLQ-OES18
[40]	Esophageal cancer patients from the Department of Thoracic Surgery of Zhongshan Hospital	Esophagus	SF-36
[41]	Head and neck cancer patients from Royal Brisbane and Women’s Hospital	Oral cavity, oropharynx, nasopharynx, hypopharynx, larynx, other	EORTC QLQ H&N35; EORTC QLQ-C30
[42]	Cancer patients from the Nutritional Counselling Centre Copernicus in Gdansk and the Department of Clinical Nutrition and Dietetics from the Medical University of Gdansk	Cranium and face, gums, tongue, sinus, throat, tonsil, esophagus, lung, stomach, pancreas	WHOQOL-BREF
[43]	Patients who underwent enhanced recovery after esophagectomy at the Department of Cardiothoracic Surgery, Jinling Hospital	Esophagus	EORTC QLQ-C30

COOP–WONCA, Dartmouth Primary Care Cooperative Information Project/World Organization of National Colleges, Academies, and Academic Associations of General Practice/Family Physicians; EORTC QLQ-C30, European Organization for the Research and Treatment of Cancer Quality of Life Questionnaire Instrument 30 items; EORTC QLQ—H&N35, European Organization for the Research and Treatment of Cancer Quality of Life Questionnaire Instrument—Head and Neck 35 items; EORTC QLQ–OG25, European Organization for the Research and Treatment of Cancer Quality of Life Questionnaire–Oesophago-Gastric Disease-Specific Quality of Life Instrument 25 items; EQ-5D, EuroQol 5-Dimensional Scale; EQ-5D-3L, EuroQol 5-Dimensional Scale with three levels; EQ-5D VAS, EuroQol 5-Dimensional Visual Analogue Scale; FAACT, Self-Administrated Functional Assessment of Anorexia/Cachexia Therapy questionnaire; FACT—G, Functional Assessment of Cancer Therapy Scale—General; FACT—O, Functional Assessment of Cancer Therapy Scale—Ovarian; MFI-20, Multidimensional Fatigue Inventory 20 items; PGWBI, Psychological General Well-Being Index; QLQ-OES18, Esophagus Specific Health-Related Quality of Life Questionnaire 18 items; SF-36, Short-Form-36 Health Survey; WHOQOL–BREF, World Health Organization Quality of Life assessment questionnaire–BREF.

**Table 5:** The basic characteristics of the groups studied within the studies included in the systematic review.

Ref.	No. of Participants (Females)	Age (Mean/Median Years with SD/Range)	Inclusion and Exclusion Criteria
[28]	31 (15)	Mean of 56.6–61.4, depending on group	Inclusion: histologically proven squamous cell carcinoma of the oral cavity, larynx, oropharynx, or hypopharynx; preoperative weight loss >10%; required major ablative surgery and eligibility for surgery Exclusion: received other investigational drugs or steroids; suffered from renal insufficiency, hepatic failure, any genetic immune disorders; confirmed diagnosis of acquired immunodeficiency syndrome (AIDS); lack of knowledge of the Dutch language
[29]	80 (27)	Mean of 62–63, depending on group	Inclusion: upper gastrointestinal tract cancer; major resective surgical procedures in the upper gastrointestinal tract Exclusion: impaired renal or hepatic function; disseminated malignant disease; ongoing corticosteroid treatment
[30]	33 (9)	60 (46–80)	Inclusion: head or neck cancer; radical (chemo)radiation treatment; patients defined as those who would probably require enteral feeding Exclusion: verified as those who did not require enteral feeding
[31]	100 (33)	Lack of data	Inclusion: stage 2 or stage 3 of squamous cell carcinoma of the head and neck; scheduled either for radical surgery with adjuvant radiotherapy (RT), chemo–RT, or for concurrent chemo- and radiation therapy Exclusion: patients with early–stage 2 of head and neck cancer
[32]	134 (43)	Mean of 60–63, depending on group	Inclusion: newly diagnosed, untreated, pharyngeal, or oral cancer, or malignant neck nodes with unknown primary in stage 3 or 4 Exclusion: treated with palliative intent; unable to answer quality of life questionnaires; not capable of following the study protocol; no possibility of having a percutaneous endoscopic gastrostomy (PEG) inserted due to previous abdominal surgery
[33]	127 (39)	Mean of 60–63, depending on group	Inclusion: newly diagnosed, oral, or pharyngeal cancer, or neck lymph node metastases with unknown primary in stage 3 or 4 Exclusion: palliative treatment; difficulties in following the protocol; participation in another clinical study
[34]	134 (43)	Mean of 60–63, depending on group	Inclusion: newly diagnosed, untreated, pharyngeal, or oral cancer, or malignant neck nodes with unknown primary in stage 3 or 4; patients surviving from [32] Exclusion: treated with palliative intent; unable to answer quality of life questionnaires; not capable of following the study protocol; no possibility of having a percutaneous endoscopic gastrostomy (PEG) inserted due to previous abdominal surgery
[35]	41 (5)	63.8 ± 8.3 *	Inclusion: confirmed diagnoses of esophageal or gastric cancer; elective esophagectomy, or total gastrectomy with the placement of feeding jejunostomy tube Exclusion: undergoing subtotal gastrectomy
[36]	109 (109)	Mean of 61.8–63.7, depending on group	Inclusion: adult females; suspected or proven advanced epithelial ovarian cancer, primary peritoneal cancer, or fallopian tube cancer; required planned upfront or interval cytoreductive surgery; signs of moderate or severe malnutrition (Patient-Generated Subjective Global Assessment (PG-SGA) category B or C); medically fit for cytoreductive surgery Exclusion: other cancers; recurrent epithelial ovarian cancer; contraindications to enteral feeding such as ileus, gastrointestinal ischemia, bilious or persistent vomiting, or mechanical obstruction; positive urine pregnancy test; unfit for surgery
[37]	90 (40)	62.5 ± 5.3	Inclusion: gastric cancer diagnoses confirmed by preoperative pathological study; no metastasis; no immunosuppressants and corticosteroid therapy within one month before surgery; transfusion therapy not used; blood loss < 400 mL during surgery Exclusion: history of hyperthyroidism, diabetes mellitus, and other metabolic diseases; dysfunction of heart, kidney, or liver; preoperative history of chemotherapy and radiotherapy; history of asthma and drug allergies; immune dysfunction or systemic infection; severe acid-base imbalance and water-electrolyte imbalance
[38]	79 (30)	Median of 67–69, depending on group	Inclusion: adult; documented cancer of the upper gastrointestinal tract (esophagus, stomach, pancreas, biliary tract); candidate for major elective surgery; preoperative nutritional risk score ≥3 (NRS 2002 tool) Exclusion: Karnofsky index < 60; renal insufficiency (ongoing hemodialysis or plasma creatinine > 3 mg/dl); respiratory insufficiency (arterial blood PaO2 < 70 mmHg); American Society of Anaesthesiology score 4–5; ChildePugh liver function class C; short bowel syndrome; pregnancy; the need for emergency; palliative surgery; foreign residence; residents in an Italian region with no specific regulation for home enteral nutrition; unable to be regularly followed-up
[39]	44 (12)	Median of 64–65, depending on group	Inclusion: adult; patients undergoing upper gastrointestinal surgery for cancer; jejunostomy feed used postoperatively without complication Exclusion: participating in another trial; oral intake at hospital discharge of >90% of requirements; if felt that they or their carers would not cope with home tube feeding; very low (<18 kg/m^2^) or high (>35 kg/m^2^) pre-operative Body Mass Index (BMI)
[40]	73 (23)	Mean of 53.2–58.3, depending on group	Inclusion: adults; scheduled esophagectomy for esophageal cancer Exclusion: age > 75 years; Body Mass Index (BMI) < 18 kg/m^2^; BMI > 30 kg/m^2^; contraindications for enteral nutrition or parenteral nutrition; preoperative initiation of enteral nutrition or parenteral nutrition; ongoing infections; preexisting organ failure (e.g., renal dysfunction required dialysis; non-compensatory chronic obstructive pulmonary disease); treatment with high doses of steroids; severe metabolic abnormalities (e.g., diabetes, hyperthyroidism/hypothyroidism); chylothorax developed; could not tolerate programmed enteral feeding
[41]	131 (16)	60.5 ± 10.1	Inclusion: adults; head or neck cancer; referred for a prophylactic gastrostomy before treatment Exclusion: pregnancy; cognitively impaired; intellectual disability or mental illness; planned for non-curative intent treatment; diagnosed as severely or moderately malnourished with significant dysphagia requiring a liquid or pureed texture modified diet
[42]	35 (8)	Mean of 60–61.1, depending on group	Inclusion: adults; the presence of cancer; artificial access to the alimentary tract (nasogastric tube, gastrostomy, percutaneous endoscopic gastrostomy, jejunostomy, micro jejunostomy); qualification for home enteral nutrition Exclusion: patients requiring home parenteral nutrition; not being able to attend the visit in the study center
[43]	50 (15)	Mean of 62.04–64.58, depending on group	Inclusion: adults; referred electively for management of nonmetastatic esophageal cancer Exclusion: American Society of Anesthesiologists physical status classes 4 and 5; cardiac failure (New York Heart Association functional classes III and IV); acute or unstable cardiac conditions (e.g., unstable angina or symptomatic severe aortic stenosis); chronic obstructive pulmonary disease (forced expiratory volume in the first second of expiration <60% predicted); physical conditions that contraindicate exercise or oral nutrition; inability to swallow; the presence of feeding jejunostomy; end-stage kidney or liver disease; psychosis

* data provided on request.

**Table 6:** The basic characteristics of the nutritional interventions associated with enteral nutrition applied within the studies included in the systematic review.

Ref.	Applied Enteral Nutrition (Studied Group/Studied Groups/Control Group)	Study Duration (Enteral Nutrition)	Other Information about Nutrition
[28]	(1) no preoperative and standard postoperative tube-feeding vs. (2) standard preoperative and postoperative tube-feeding vs. (3) arginine-supplemented preoperative and postoperative tube-feeding (41% of casein proteins replaced by arginine)	7–10 days before surgery and 14 days after surgery	Energy value of 150% of basal energy expenditure
[29]	(1) postoperative oral supportive nutrition vs. (2) specialized enteral nutrition (1000 kcal/day) vs. (3) specialized parenteral nutrition (900 kcal/day)	Maximum 12 months after surgery until the preoperative weight was reached	Oral supportive nutrition
[30]	(1) nasogastric (NG) tube feeding vs. (2) percutaneous endoscopic gastrostomy (PEG) tube feeding	(1) median of 66 days (23–136 days) (2) median of 139 days (56–488 days)	Energy value of 50–100% of energy requirement (median of 100%)
[31]	(1) percutaneous endoscopic gastrostomy (PEG) tube feeding vs. (2) nasogastric (NG) tube feeding	6 weeks	Lack of data
[32]	(1) tube feeding initiated if the oral intake became inadequate (>1 kg weight loss) vs. (2) standard care (nutritional advice and enteral tube feeding when necessary)	24 months	Energy value calculated as 30 kcal/kg body weight/day and protein intake need calculated as 1.2–1.5 g/kg body weight/day
[33]	(1) prophylactic percutaneous endoscopic gastrostomy (PEG)—tube feeding initiated if the oral intake became inadequate (>1 kg weight loss) vs. (2) standard care (nutritional advice and enteral tube feeding when necessary)	24 months	Energy value calculated as 30 kcal/kg body weight/day (for Body Mass Index (BMI) > 25 kg/m^2^—ideal weight for BMI of 25 kg/m^2^ used for calculation) and protein intake need calculated as 1.2–1.5 g/kg body weight/day
[34]	(1) tube feeding initiated if the oral intake became inadequate (>1 kg weight loss) vs. (2) standard care (nutritional advice and enteral tube feeding when necessary)	24 months of study as reported by [32], followed-up after 8 years	Energy value calculated as 30 kcal/kg body weight/day and protein intake need calculated as 1.2–1.5 g/kg body weight/day
[35]	(1) overnight jejunostomy feeding via an electronic pump vs. (2) routine clinical care (discontinuation of jejunostomy feeds on the day of hospital discharge but provided when necessary—if weight loss >5% from baseline, reduced functional status, or estimated oral calorie intake <33% of requirements)	6 weeks	Energy value and protein intake of 50% of energy and protein requirement to be provided by supplementary jejunostomy; food fortification and the use of prescribable nutritional supplements for all patients
[36]	(1) nasojejunal tube feeding until the participant was able to maintain an adequate oral intake (65–75% of daily nutritional requirements) vs. (2) standard diet	30 days	Nasojejunal tube feeding with standard fiber-containing, high-protein enteral nutrition formula (20% protein, 30% fat, 50% carbohydrate) to provide 30 kcal/kg body weight/day
[37]	(1) Low-nitrogen and low-calorie parenteral combined with enteral nutrition and supplemented by targeted nursing intervention vs. (2) total parenteral nutrition (TPN)	Lack of data	(1) Parenteral nutrition: 20 kcal/kg body weight/day, with nitrogen of 0.09–0.11 g/kg body weight/day and non-protein calorie of 16–20 kcal/kg body weight/day (2) Total parenteral nutrition: 30–35 kcal/kg body weight/day, with nitrogen of 0.19–0.21 g/kg body weight/day and non-protein calorie of 28–32 kcal/kg body weight/day
[38]	(1) oral intake accompanied by home enteral nutrition (discontinuation of enteral nutrition after 2 months from discharge, if weight gain ≥5% was reported and oral diet was regular and adequate) vs. (2) oral intake only with oral nutritional supplements if needed (enteral nutrition allowed, but not before 2 months from discharge if a weight loss ≥5% was reported)	2 months	Energy value of enteral nutrition to cover basal energy requirement (12–20% protein, 25–35% fat, 50–60% carbohydrate)
[39]	(1) jejunal feeding vs. (2) no post-operative jejunal feeding after discharge	6 weeks	Jejunal feeding of 600 kcal/day; both groups offered oral nutritional supplements to take at home
[40]	(1) enteral nutrition and supplementary parenteral nutrition (to meet energy requirements) vs. (2) enteral nutrition	9 days	Both groups received parenteral minerals (potassium, phosphate, calcium, and magnesium), vitamins, and trace elements after surgery; parenteral calories from fat (30% of calories) and carbohydrates (70%); target protein intake in the group receiving enteral nutrition and supplementary parenteral nutrition—1.5 g/kg fat-free mass/day; insulin continuously infused to maintain a blood glucose concentration <10 mmol/L
[41]	(1) enteral nutrition in addition to their current oral intake immediately (600 kcal—polymeric formula with fiber and was increased as necessary) vs. (2) standard nutrition with enteral nutrition only if necessary (oral intake < 60% of estimated energy requirements or anticipated to be for >10 days, or the patient unable to maintain weight, or the significant texture modification of diet required, or increased or uncontrolled nutrition impact symptoms)	Lack of data	Both groups were encouraged to maintain oral intake as much as possible during treatment and as long as it remained safe to do so
[42]	(1) standard enteral formula vs. (2) standard enteral formula with *Lactobacillus plantarum* 299v	4 weeks	Standard normo-caloric enteral formula without additional fiber
[43]	(1) enhanced nutrition support (additional nutrition support via oral intake or jejunostomy tube: 7 preoperative days—500–1000 kcal/day and after discharge—500 kcal/day) vs. (2) conventional nutrition (additional nutrition support via oral intake or jejunostomy tube only if NRS2002 score ≥3 during 7 preoperative days—500–1000 kcal/day)	1 month	Both groups oral intake of semi-liquid diet

**Table 7:** The results and conclusions associated with the effect of the applied nutritional intervention on the quality of life within the studies included in the systematic review.

Ref.	Observations	Conclusions
[28]	Between baseline and the day before surgery, both preoperatively fed groups revealed a positive change for the dimensions of physical and emotional functioning and dyspnea (with significance in group II, *p* = 0.050, 0.031, 0.045 respectively). Group III showed a negative change in appetite (*p* = 0.049). Between baseline and 6 months after surgery, there were no differences between Group I and both pre-fed groups. There were no differences in favor of Group III compared to Group II.	Enteral nutrition improves quality of life of severely malnourished head and neck cancer patients in the period preceding surgery. No benefit of preoperative enteral feeding on quality of life could be demonstrated 6 months after surgery.
[29]	Parenteral feeding was associated with the highest rate of nutrition-related complications, whereas enteral feeding reduced quality of life most extensively.	After major surgery, specialized supportive enteral and parenteral nutrition are not superior to oral nutrition only when guided by a dietitian.
[30]	Nutritional support with both tubes was good. There were no significant differences in patients’ assessment of their overall quality of life.	There is no evidence to support the routine use of percutaneous endoscopic gastrostomy tubes over nasogastric tube in the studied patient group.
[31]	There was a statistically significant difference between the two groups in patients’ quality of life scores and complications.	Percutaneous endoscopic gastrostomy is more efficacious for quality of life than nasogastric tube as a channel for nutrition in advanced head and neck cancer patients over a short duration.
[32]	After 6 months, quality of life was significantly better and the weight loss was significantly less in the study group.	Prophylactic percutaneous endoscopic gastrostomy was associated with significantly earlier start and longer use of enteral nutrition, fewer malnourished patients over time, and improved quality of life at 6 months posttreatment start.
[33]	Both groups lost weight the first six months due to insufficient energy intake and used enteral nutrition as their main intake source; no significant differences between groups were found. Problems with dysphagia were vast during the 6 months. Oral intake was the major energy source after 1 year.	Head and neck cancer patients need nutritional support and enteral feeding for a long time period during and after treatment due to insufficient energy intake. A prophylactic percutaneous endoscopic gastrostomy did not significantly improve the enteral intake probably due to treatment side effects.
[34]	There was no significant difference in swallowing function between the groups after 12 months, 24 months, and 8 years, the oral intake scale, tube dependence, esophageal intervention, and overall survival.	A prophylactic percutaneous endoscopic gastrostomy tube can be used without an increased risk of long-term dysphagia in patients with head and neck cancer.
[35]	The global quality of life scores deteriorated in both groups after surgery, but approached baseline levels in both groups by six months.	The study demonstrated that home enteral feeding by jejunostomy was feasible, safe, and acceptable to patients and their carers.
[36]	No significant difference in quality of life was found between the groups at any time point.	Early enteral feeding did not significantly improve patients’ quality of life compared to standard of care but may improve nutritional status.
[37]	A low-nitrogen and low-calorie parenteral nutrition combined with enteral nutrition can effectively improve the postoperative quality of life.	A low-nitrogen and low-calorie parenteral nutrition combined with enteral nutrition can be suitable for clinical application.
[38]	After 2 months, patients on home enteral nutrition maintained their mean body weight, while patients in the nutritional counselling group showed a weight loss of 3.6 kg. Patients supported on home enteral nutrition had a higher chance to complete chemotherapy as planned (48% versus 34%). Quality of life was not worsened by home enteral nutrition.	The study lends support to the importance of home enteral nutrition in upper gastrointestinal cancer patients, after major surgery, as it helps maintain body weight without any safety concern or detrimental impact on quality of life.
[39]	After hospital discharge, there were no differences in scores at any time point. From hospital discharge fatigue improved and plateaued at 6 weeks (*p* < 0.05 for both groups), independence at 12 weeks (*p* < 0.05 for both groups). No improvement was seen in quality of life until 24 weeks in the intervention group alone (*p* < 0.02) and not at all in the control group.	Addition of jejunal feeding is effective in providing patients with an adequate energy intake. Increased energy intake however, produced no obvious improvement in measures of fatigue, quality of life or health economics.
[40]	Scores for physical functioning (71.5 ± 24.3 vs. 60.4 ± 27.4, *p* < 0.05) and energy/fatigue (62.9 ± 19.5 vs. 54.2 ± 23.5, *p* < 0.05) were higher in the enteral + parenteral nutrition group 90 days following surgery.	Early use of supplemental parenteral nutrition to meet full calorie requirements of patients who underwent esophagectomy led to better quality of life 3 months after surgery.
[41]	No differences were found for quality of life or clinical outcomes.	The early intervention did not improve outcomes, but poor adherence to nutrition recommendations impacted on potential outcomes.
[42]	The improvement of quality of life was observed in both groups; however, with no statistically significant differences between the analyzed groups (*p* > 0.05).	Lp299v may reduce the gastrointestinal symptoms related to enteral nutrition; notwithstanding, the improvement of quality of life may be the result of enteral nutrition rather than the effect of administration of Lp299v.
[43]	Enhanced nutritional support improved the quality of life of patients in physical function (75.13 ± 9.72 vs. 68.33 ± 7.68, *p* = 0.009) and fatigue symptom (42.27 ± 9.93 vs. 49.07 ± 11.33, *p* = 0.028) compared to conventional nutritional support.	This pilot study demonstrated that an enhanced nutritional support pathway including extended preoperative nutritional support and home enteral nutrition was feasible, safe, and might be beneficial to patients who underwent enhanced recovery after esophagectomy.

**Table 8:** The summary of conclusions from the studies comparing patients treated with and without enteral nutrition included to the systematic review accompanied by the Newcastle–Ottawa Scale (NOS) score.

	Conclusion about Influence of Enteral Nutrition on the General Quality of Life *	Disease Location	Quality of the Study Based on the NOS Score **
[28]	Results supporting enteral nutrition	Oral cavity, oropharynx, hypopharynx, larynx, other	7
[32] ***	Results supporting enteral nutrition	Oropharynx, oral cavity, hypopharynx, nasopharynx, other	7
[33]	Results not supporting enteral nutrition	Oral cavity, pharynx, neck lymph node	7
[34] ***	Results supporting enteral nutrition	Oropharynx, oral cavity, hypopharynx, nasopharynx, other	7
[35]	Inconclusive results	Esophagus, stomach	6
[36]	Results supporting enteral nutrition	Ovary	7
[38]	Results supporting enteral nutrition	Esophagus, pancreas, stomach, biliary tract	5
[39]	Results supporting enteral nutrition	Esophagus, stomach	6
[41]	Results not supporting enteral nutrition	Oral cavity, oropharynx, nasopharynx, hypopharynx, larynx, other	9
[43]	Results supporting enteral nutrition	Esophagus	6

* in case of no influence on the psychological measures of the quality of life, its influencing factors are taken into account (e.g., malnutrition, complications, etc.); ** total score of: 0–3—very high risk of bias, 4–6—high risk of bias, 7–9—low risk of bias; *** the same cohort studied in [32,34].

**Table 9:** The summary of conclusions from the studies comparing patients treated with enteral and parenteral nutrition, with various methods of enteral nutrition, and with enteral nutrition of various contents, included in the systematic review accompanied by the Newcastle–Ottawa Scale (NOS) score.

Ref.		Conclusion about Influence of Enteral Nutrition on the General Quality of Life *	Disease Location	Quality of the Study Based on the NOS Score **
Patients treated with enteral and parenteral nutrition	[29]	Specialized enteral/parenteral nutrition not superior to supervised oral supportive nutrition	Esophagus, stomach, pancreas	7
	[37]	Enteral + parenteral nutrition superior to parenteral nutrition	Stomach	4
	[40]	Enteral + parenteral nutrition superior to enteral nutrition	Esophagus	7
Patients treated with various methods of enteral nutrition	[30]	Percutaneous Endoscopic Gastrostomy and Nasogastric Tube—comparable	Oral cavity, oropharynx, nasopharynx, hypopharynx, larynx, other	5
	[31]	Percutaneous Endoscopic Gastrostomy superior to Nasogastric Tube	Hypopharynx, larynx, nasopharynx, oropharynx	6
Patients treated with enteral nutrition of various content	[42]	No effect of including Lp299v to enteral nutrition	Cranium & face, gums, tongue, sinus, throat, tonsil, esophagus, lung, stomach, pancreas	6

* in case of no influence on the psychological measures of the quality of life, its influencing factors are taken into account (e.g., malnutrition, complications, etc.); ** total score of: 0–3—very high risk of bias, 4–6—high risk of bias, 7–9—low risk of bias.
